# Extrinsic and intrinsic factors influencing the emergence and return of the Asian particolored bat *Vespertilio sinensis* to the summer roost

**DOI:** 10.1002/ece3.8890

**Published:** 2022-05-13

**Authors:** Lei Feng, Hexuan Qin, Jingjing Li, Xin Li, Jiang Feng, Tinglei Jiang

**Affiliations:** ^1^ 47821 Jilin Provincial Key Laboratory of Animal Resource Conservation and Utilization Northeast Normal University Changchun China; ^2^ Key Laboratory of Vegetation Ecology of Education Ministry Institute of Grassland Science Northeast Normal University Changchun China; ^3^ College of Animal Science and Technology Jilin Agricultural University Changchun China

**Keywords:** abiotic factors, bats, biotic factors, circadian rhythm, predation, reproduction status

## Abstract

Circadian rhythms play a crucial role in the health and survival of organisms. However, little is known concerning how intrinsic and extrinsic factors affect animal daily rhythms in the field, especially in nocturnal animals. Here, we investigated the first emergence, mid‐emergence, and return times of *Vespertilio sinensis*, and also integrated environmental conditions (temperature, humidity, and light intensity) and biotic factors (reproductive status and predation risk) to determine causes of variation in the activity rhythms of the bats. We found that variation in the first emergence time, the mid‐emergence time, and the final return time were distinct. The results demonstrated that the emergence and return times of bats were affected by light intensity, reproductive status, and predation risk in a relatively complex pattern. Light intensity had the greatest contribution to activity rhythms. Moreover, we first investigated the effects of actual predators on the activity rhythms of bats; the results showed that the mid‐emergence time of bats was earlier as predators were hunting, but the final return time was later when predators were present. Finally, our results also highlighted the importance of higher energy demands during the lactation in bats to variation in activity rhythms. These results improve our understanding of the patterns and causes of variation in activity rhythms in bats and other nocturnal animals.

## Graphical abstract

Circadian rhythm is an adaptive life history characteristic of an organism. For example, human beings are active during the day and rest at night, which is the behavior manifestation of circadian rhythm. Animals’ circadian rhythms are influenced by a variety of factors such as light, temperature and humidity, reproduction, and predators. So, we wanted to know what would affect the circadian rhythms of Asian particolored bats, which is a small bat species found in East Asia.

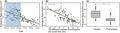

## 1 INTRODUCTION

Circadian rhythms are endogenous and common patterns in the activity of organisms, from prokaryotes to eukaryotes, over periods of about 24 h (Bell‐Pedersen et al., 2005; McClung, 2000). Circadian rhythms are crucial to the health and survival of organisms, as they allow organisms to anticipate the daily environmental changes and implement appropriate strategies (Caravaggi et al., 2018; Yerushalmi & Green, 2009). Global climate change is already impacting the activity rhythms of many animals (Levy et al., 2018). Environmental conditions and biotic factors have been considered drivers of variation in the circadian activity rhythms of many vertebrates (Pita et al., 2011). However, little is known concerning how intrinsic and extrinsic factors influence animal daily rhythms in the field (Quaglietta et al., 2018).

Activity rhythms are influenced by many abiotic factors, including light, moon phase, temperature, and humidity (Tester & Figala, 1990). Light is an important modulator of organisms’ circadian rhythms (LeGates et al., 2014). For example, some nocturnal animals may decrease their activity during a full moon; this is known as “lunar phobia” (Mougeot & Bretagnolle, 2000; Saldana‐Vázquez & Munguia‐Rosas, 2013). Extreme weather conditions could affect animals’ activity patterns to avoid overheating or hyperthermia, causing animals to reduce their activity during high‐ or low‐temperature conditions (Foà & Bertolucci, 2001; Frick et al., 2012; Speakman et al., 1994). Additionally, drought could affect animal and plant populations and constrain the activity of prey, thereby increasing the competition for food (Frick et al., 2012).

In addition to abiotic factors, biotic factors such as predation risk and reproductive status also may influence animals’ activity rhythms (Arndt et al., 2018). Predation risk is a strong selection pressure affecting activity rhythms (John, 2013; Lima & O’Keefe, 2013). Some animals, such as bats, face a tradeoff between predation risk and energy requirements when they decide to begin an activity during the period of day‐to‐night transition (Arndt et al., 2018; Shiel & Fairley, 1999; Shuert et al., 2020). During lactation, reproductive female bats have higher energy requirements than other individuals (Lučan, 2009; Shiel & Fairley, 1999); thus, they must balance increases in foraging time while maximizing food availability and minimizing the possibility of being captured by predators (Caro, 2005). In general, an earlier start to foraging activity may be beneficial (Pavey et al., 2001), but may also expose animals to a higher predation risk (Fenton et al., 1994; Jones & Rydell, 1994). Thus, a successful strategy requires the optimal adjustment between predation pressure and foraging activity in order to maximize energy intake (Caro, 2005). However, it remains unclear how animals adjust activity rhythms based on predation risk and energy requirement, especially during the lactation.

Bats are an excellent system for investigating questions concerning the effects of abiotic and biotic factors on activity rhythms for the following reasons. First, bats are active almost exclusively during the night (Rydell & Speakman, 1995; Speakman, 1991). In this case, bats are sensitive to environmental changes, especially to light (Voigt & Kingston, 2016). Second, bats normally start nighttime activity for foraging after sunset, but not in total darkness (Lee & McCracken, 2001; Pavey et al., 2001). Like other animals, bats would benefit from an earlier emergence that would allow the bats to follow the activity peaks of some insects during dusk (Rydell et al., 1996). Moreover, although earlier onset of activity would increase the amount of foraging time at dusk and dawn (Pavey et al., 2001), it would increase the chance of bats encountering diurnal predators that remain active around sunset, or even during dusk (Fenton et al., 1994; Lima & O’Keefe, 2013). Thus, it is necessary to clarify the effects of risk of long‐term natural predation and energy requirements for reproduction on the activity rhythms of bats. Finally, previous studies have focused on the effects of environmental factors such as temperature (Arndt et al., 2018; Irwin & Speakman, 2003), cloud cover (Arndt et al., 2018; Lee & McCracken, 2001; McAney & Fairley, 1988), heavy rain (McAney & Fairley, 1988), and light (Russo et al., 2007) on the emergence of bats, but little is known concerning the impact factors affecting the return times of bats, or how interactions of abiotic and biotic factors influence the activity rhythms of bats.

In this study, we monitored the emergence and return times of the Asian particolored bat (*Vespertilio sinensis*), and integrated environmental conditions (temperature, humidity, and light intensity) and biotic factors (reproductive status and predation risk) to determine the sources of variation in activity rhythms of bats. We tested three hypotheses. First, we hypothesized that light intensity may play a pivotal role in influencing the emergence and return times of *V*. *sinensis*. Second, since bats have greater energy requirements during lactation, we hypothesized that *V*. *sinensis* would depart from the roost earlier during lactation than during postlactation. Third, since bats should avoid predators, we hypothesized that *V*. *sinensis* would depart from the roost at dusk later, but return to the roost earlier at dawn in the presence of diurnal predators (e.g., falcons).

## 2 MATERIALS AND METHODS

### 2.1 Study site and species

The study was conducted at a highway bridge (126°57′26′′ E, 45°32′52′′ N) in Acheng District, Heilongjiang Province, northeast China. This was a reinforced concrete bridge with a length of more than 500 m. The bridge was composed of fourteen archways that were defined as the spaces surrounded by two beams (Figure S1a). Every archway included about twelve crevices where bats would roost (Figure S1b). The bridge is located at the edge of the city; there is only a small amount of vegetation around the bridge, but there are many artificial buildings (Figure S1c,d). During the daytime in August 2020, we stood on scaffolding and counted bats using flashlights (Figure S2d). To reduce human disturbance to the bats, only three arches were investigated. In addition, it was difficult to build stable scaffolding under most of the arches. Bats roosted in the crevices of 14 arches on the bridge; hence, we estimated that there was an average of 450 bats per arch, and that this bat population numbered about 6300 individuals. In summer, female *V*. *sinensis* roost and breed offspring here, forming a nursery (Wang et al., 2020). The population of bats arrives in the summer roost in mid‐June and leaves the summer roost by the end of September. We found that the Amur falcon (*Falco amurensis*) started to prey on *V*. *sinensis* during mid‐August in 2018; this provided an opportunity to determine the effects of predation on activity rhythms of *V*. *sinensis*.

### 2.2 Monitoring of activity rhythms

Roost emergence times were recorded from early or mid‐July (July 10th in 2019, July 7th in 2020, and June 30th in 2021) to late August or early September (September 10th in 2019, September 5th in 2020, and August 27th in 2021) from 2019 to 2021. The final return times were recorded from early or the middle of July to late August, or from early September, in 2019 and 2020. The monitoring was stopped when it was raining heavily. We arrived at the roost of bats one hour before sunset to record the first emergence time, which was defined as the first bat to emerge and leave the roost. Additionally, a previous study showed that first emergence time was a statistically poorer predictor parameter for describing the emergence time of the whole roost than the median emergence time (Bullock et al., 1987). Therefore, we also included the median emergence time as a descriptive parameter for emergence time of *V*. *sinensis*. We defined the median of the first emergence time and the end time of emergence as the mid‐emergence time. When there were no bats flying out for five minutes, we considered the emergence activities of the bats to have finished.

During lactation, we found adult female bats returning to the daytime roost about three hours after first emergence time, and we also found bats leaving the roost. We suspected that female bats were returning to the roost to nurse their offspring during the night. When the juveniles could fly, they would fly around in the roost at night, but would not leave the roost; we suspected that this behavior might take place in order to enhance their flight ability. These two phenomena would make it difficult for us to confirm return parameters of bats (such as start time of return and duration of return). Moreover, *F*. *amurensis* only hunted bats near the bats’ roost during the final return period of *V*. *sinensis* at dawn. Thus, we only considered the last return time as the return parameter of bats observed in the dawn dataset. We arrived at the roost of the bats an hour and a half before sunrise the next day after recording the dusk data to collect the data of final return times of the bats. The final return time was observed and recorded when the last bat returned to the day roost.

### 2.3 Collection of environmental data

We recorded the sunrise and sunset times through the network https://richurimo.51240.com/ based on the longitude and latitude of the roost of *V*. *sinensis*. Here, the time was accurate to the nearest second. One researcher recorded the first emergence time and the final return time. Another researcher simultaneously measured the light intensity (lux), air temperature (°C), and humidity (%) using a Five in One Multifunctional Environment Meter (HT‐8500, HCJYET). The environmental data were measured at the same location on the highway bridge without objects blocking natural light or external artificial light sources. The final return times were recorded in 2019 and 2020. Additionally, we recorded the light intensity of natural light every 30 s in 2019 to 2021. Then, we determined the light intensity of the median of emergence events by selecting the point in time closest to the median of emergence.

### 2.4 The division of reproductive status

In order to investigate the effects of reproductive state on activity rhythms of *V*. *sinensis*, we divided the monitoring period into lactation and postlactation. During the lactation (from July 1st to July 31st), juvenile bats were observed in early and mid‐July (Figure S3), and volitant subadult bats were observed in late July, but they did not leave the daytime roost to forage. During postlactation (from August to early September), there were very few subadult bats flying near the daytime roost, and there were almost no subadult bats flying near the daytime roost at night by August 10th. After mid‐August, all subadults had grown and emerged from the roost to forage at night.

### 2.5 Predation risk assessment

In order to explore the effects of predators on the activity rhythms of *V*. *sinensis*, we defined predation risk as the presence or absence of predators. Every dusk, before the start of emergence of *V*. *sinensis*, if the falcons appeared near the daytime roost of bats and hunted emerged bats, this was considered as the presence of predators (high predation risk) when we were monitoring the emergence (Figure S2a–c). The observation sites were located above an overpass, surrounded by only a small amount of vegetation and low residential buildings. This allowed us to quickly observe *F*. *amurensis* and determine predation risk. We also observed that *F*. *amurensis* simply flew over the roost of bats, without waiting for the bats. Additionally, if the bats emerged from the roost too late, the predators would give up waiting for bats before the bats started to emerge. The same phenomenon happened in the dawn; the Amur falcons were unable to catch the bats if the bats return too early, and they would give up waiting for the bats. Therefore, if there were no *F*. *amurensis*, or if *F*. *amurensis* only flew across the roost without waiting for emerged bats, this was considered as the absence of predators (low predation risk). Since *F*. *amurensis* arrived at the bats’ roost to hunt bats every day during lactation and part of postlactation of the bats, and sometimes, *F*. *amurensis* were present after mid‐emergence. In this case, we considered predation risk as “predators were hunting” or “predators were not hunting” when determining mid‐emergence time. During the monitoring period, we did not observe any other potential diurnal avian predator near the roost. Therefore, we believed that only *F*. *amurensis* exerted diurnal predation pressure on *V*. *sinensis*.

### 2.6 Statistical analysis

In this study, we used differences between sunset time and first emergence time of bats as well as the difference between sunset time and mid‐emergence time as two variables to quantify the time of first emergence and median emergence of bats. Moreover, we also used differences between sunrise and final return time as a variable to quantify the final return time of bats. The three variables were tested for normality by Shapiro–Wilk tests. We found that only differences between sunrise and final return time did not follow a normal distribution (*p* < .001). In this case, the logarithmic transformation was used to make the data meet a normal distribution (*p* = .407).

In order to state and use statistics more easily, we used the following abbreviations for independent variables measured at dusk, which were used to construct models to test the effects of these variables on emergence times of bats: HFE, humidity at first emergence of bats; HME, humidity at median emergence of bats; TFE, temperature at first emergence of bats; TME, temperature at median emergence of bats; SST, sunset time; LISS, light intensity of sunset; LIFE, light intensity of first emergence; LS, lactation stages (lactation or postlactation); PAPDD, presence or absence of predators during dusk; LME, light intensity of mid‐emergence; PHNHM, predators were hunting or not hunting in mid‐emergence. Additionally, variables measured at dawn were considered as independent variables for the analysis of the effects of factors on final return events: HFR, humidity at final return of bats; TFR, temperature at final return of bats; SRT, sunrise time; LISR, light intensity at sunrise; LS, lactation stages (lactation or postlactation); PAPDA, presence or absence of predators during dawn; LIFR, light intensity at final return.

To test the effects of environmental factors, reproduction, and predation on the times of first emergence, mid‐emergence, and final return of *V*. *sinensis*, we selected optimized linear models using the ‘dredge’ function in the package ‘MuMin’ in R 3.5.1 (R Core Development Team, 2018). In the first model, we used the difference between sunset time and first emergence time of bats as a dependent variable; HFE, TFE, SST, LISS, LS, PAPDD, and LIFE as independent variables. In the second model, we used the difference between sunset time and mid‐emergence time as a dependent variable; HME, TME, ST, LISS, LIFE, LIME, LS, and PHNHM as independent variables. In the third model, we used the difference between sunrise time and final return time as a dependent variable; HFR, TFR, SRT, LISR, LS, PAPDA, and LIFR as independent variables. In each model, year was considered a random variable.

In order to test the multicollinearity of variables, the Variance Inflation Factor (VIF) of each predicted factor was calculated to determine which predictors could not be used for subsequent analysis. If VIF was <5, the corresponding predictor variables were included in the models (Lin & Feng, 2008). An ANOVA was used to detect whether interactions between factors in each dataset needed to be considered (Shi et al., 2010). We compared the models using the Akaike information criterion corrected for small sample size (AICc). We also calculated the Akaike weight (*w_i_*) to estimate the relative likelihood of a given model, which was then compared with other candidate models. The model with the minimum AICc and the maximum Akaike weight was the best‐fitting model. Furthermore, we calculated ΔAICc as the difference between the AICc of each model and the AICc of the best‐fitting model. If the difference of AICc (ΔAICc) between the first and the second model was greater than 2, this indicated that the first model had better explanatory power than the second model (Anderson & Burnham, 2002). If the ΔAICc between the first model and the second model was less than 2, multimodel inference was performed to determine factors significantly impacting the independent variables using the function ‘model.avg’ in the package ‘MuMIn’ (Bartoń, 2017). Additionally, we conducted a hierarchical partitioning analysis in the ‘hier.part package’ (Walsh & Nally, 2020) to estimate the independent effect of each predictor variable (Chevan & Sutherland, 1991). Additionally, in order to investigate whether there were significant differences in the number of bats successfully predated by *F*. *amurensis* between dusk and dawn, we compared the number of bats predated successfully between the two periods using a Mann–Whitney *U* test. We conducted a Chi‐square test to determine whether bats tended to start emergence before sunset during lactation.

## 3 RESULTS

### 3.1 The impact factors of first emergence time of *V. sinensis*

**FIGURE 1:**
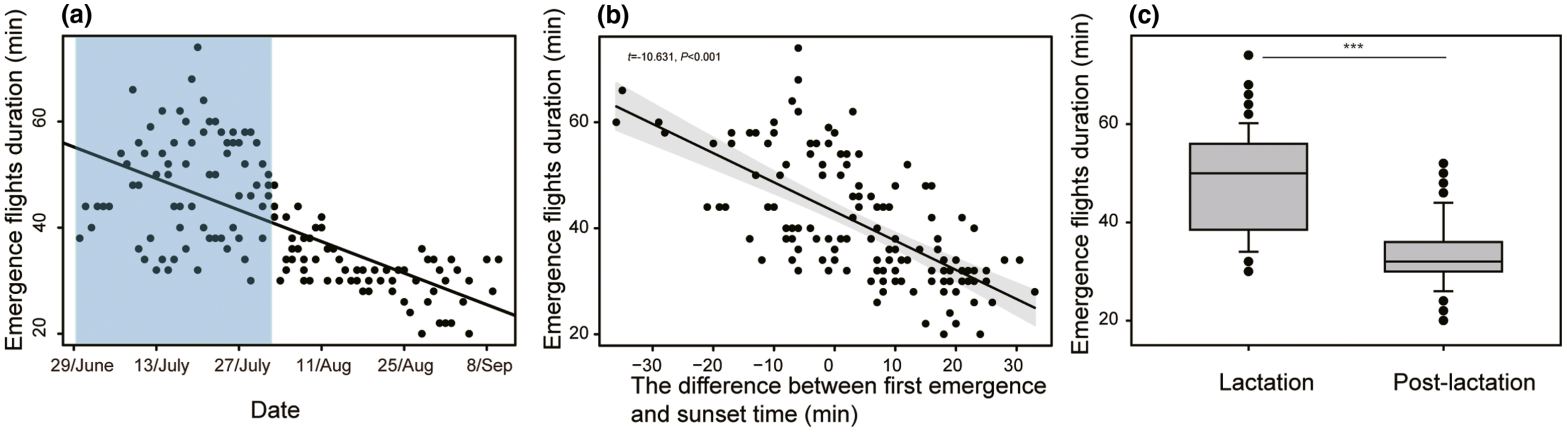
(a) The change in the duration of emergence flights of bats from 2019 to 2021, where the light blue squares indicate the lactation of *V*. *sinensis*; (b) the relationship between the duration of emergence flights of bats and the difference between first emergence of bats and sunset time, where the gray area represents the 95% CI; (c) the duration of emergence flights of bats between lactation and postlactation was significantly different, ***Means *p* < .001

A total of 140 first emergence events of *V*. *sinensis* were recorded. There were no significant differences in first emergence time among the three consecutive years (*H* = 2.804, *p* = .246). In 49 records, *V*. *sinensis* started to emerge before sunset. Differences between sunset and first emergence ranged from −36 to 33 (4.493 ± 13.231) min. Here, the negative values meant that first emergence events started before sunset. The duration of emergence was 20 to 74 (40.618 ± 11.369) min. During the data collection period of each year, the length of emergence duration gradually decreased (Figure 1a), and the emergence duration also decreased with the delay of the bats’ emergence from the daytime roost (Figure 1b). Additionally, there was a significant difference in the duration of emergence between lactation and postlactation (Figure 1c).

**FIGURE 2:**
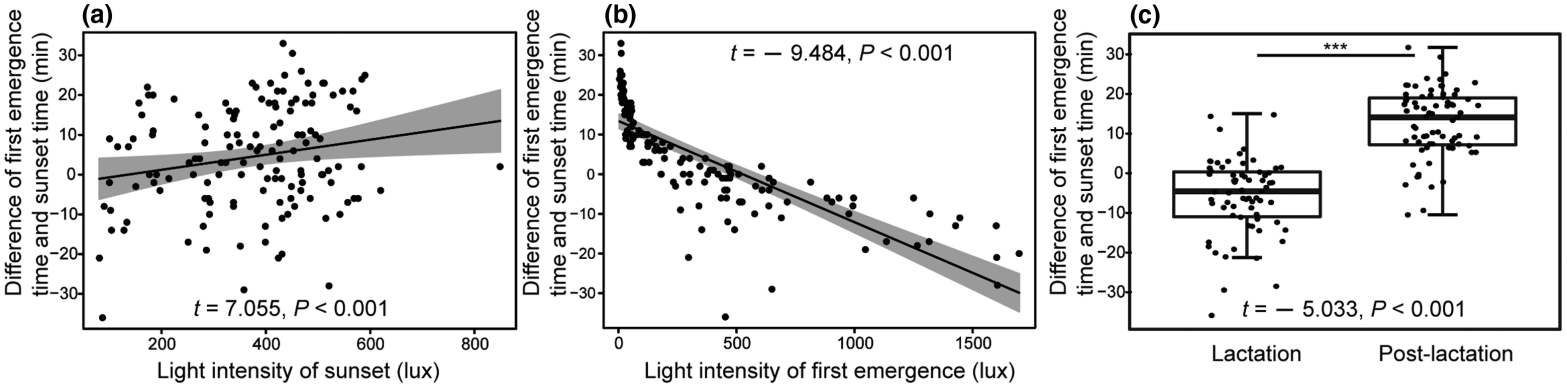
The relationships between differences (between sunset time and first emergence time) and light density of sunset (a), and light density at first emergence (b), and reproductive status (c). The gray area represents the 95% CI. ***Means *p* < .001

Our results indicated that the best model described the variation in first emergence time of *V*. *sinensis* used LIFE, LISS, LS, and PAPDA as the predictor variables. In this model, LIFE and LS, as well as PAPDA and LS, needed to be considered for interactions (Table 1). Furthermore, the LIFE, LS, LISS, and interactions between LIFE and LS were significantly associated with the first emergence time of *V*. *sinensis* (Table 2). Differences between first emergence time and sunset time were significantly and negatively associated with light intensity of first emergence (linear regression: *t* = −9.484, *p* < .001, Figure 2b). The differences between first emergence time and sunset time were significantly and positively associated with light intensity at sunset (*t* = 7.055, *p* < .001; Figure 2a). The significant differences in the first emergence time were detected between lactation and postlactation (*t* = −5.033, *p* < .001; Figure 2c). During lactation, *V*. *sinensis* started to emerge earlier from the roost than during postlactation (Figure 2c). Specifically, 65.67% of first emergence events (44/67) started before sunset during lactation. In contrast, only 6.85% of first emergence events (5/73) were observed before sunset during postlactation. A Chi‐square test of independence showed that the first emergence events had a higher probability of occurring before sunset during the lactation (χ^2^ = 26.34, *p* = 2.86 × 10^−7^). Additionally, the LIFE and LS together had significant impacts on the first emergence time of *V*. *sinensis*, but the PAPDD did not have a significant influence on the first emergence time of *V*. *sinensis* (Table 2). The results of the hierarchical partitioning analysis showed that the independent contributions of LIFE, LS, LISS, and PAPDD were 51.262%, 33.257%, 9.226%, and 6.255%, respectively.

**TABLE 1:** Candidate linear mixed models explaining the variation in first emergence time of *Vespertilio sinensis* based on environmental parameters (temperature, relative humidity, and light intensity) and biotic factors (reproductive stages and predation risk) at dusk

Model	Predictive variables	k	df	△AICc	*w_i_*
M1	LIFE, PAPDD, LS, LISS, LIFE*LS, PAPDD*LS	6	9	0.000	0.545
M2	HFE, LIFE, LS, PAPDD, LISS, LIFE*LS, PAPDD*LS	7	10	2.322	0.171
M3	LIFE, TFE, LS, LISS, PAPDD, LIFE*LS, PAPDD*LS	7	10	3.423	0.098
M4	LIFE, LS, LISS, PAPDD, LIFE*LS	5	8	4.721	0.051
M5	LIFE, LS, LISS, LIFE*LS	4	7	4.838	0.049
M6	HFE, LIFE, PAPDD, LS, TFE, LISS, LIFE*LS	7	11	6.229	0.024
M7	HFE, LIFE, PAPDD, LS, LISS	5	9	6.818	0.018
M8	HFE, LIFE, LS, LISS, LIFE*LS	5	8	6.921	0.017
M9	LIFE, TFE, PAPDD, LS, LISS, LIFE*LS	6	9	7.953	0.010
M10	LIFE, TFE, LS, LISS, LIFE*LS	5	8	8.017	0.010

Note

Models are ranked by Akaike’ s Information Criterion corrected for small sample sizes (AICc) values, from the most plausible model to the tenth most plausible model.

Abbreviations: LIFE, light intensity of first emergence of bats; LISS, light intensity of sunset; HFE, humidity as first emergence of bats; TFE, temperature as first emergence of bats; LS, lactation stages; PAPDD, presence or absence of predators during dusk.

**TABLE 2:** The parameter estimates of the best‐supported (before and including the null model) linear mixed models describing the variation in first emergence time of *Vespertilio sinensis*. The independent effects (IF) of factors on the first emergence time of *Vespertilio sinensis* using hierarchical partitioning analysis are displayed in the last column

Variable	Estimate	SE	*t* value	*p*	95% CI	IF
(Intercept)	10.491	1.774	5.914	<.001	6.905, 13.953	—
LIFE	−0.066	0.007	−9.484	<.001	−0.079, −0.052	51.262
LS	−19.580	3.891	−5.033	<.001	−27.144, −11.032	33.257
LISS	0.0241	0.003	7.055	<.001	0.017, 0.031	9.226
PAPDD	−0.575	1.373	−0.419	.676	−3.241, 2.240	6.255
LIFE*LS	0.049	0.007	6.848	<.001	0.035, 0.063	—
PAPDD*LS	5.836	3.612	1.615	.1086	−1.310, 12.982	—

**FIGURE 3:**
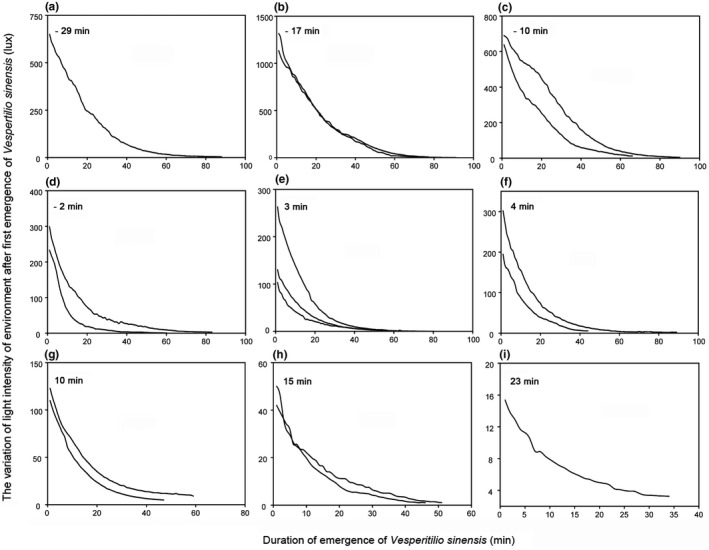
The variation in natural light intensity per unit time after the first bats emerged from their daytime roost. The scale range of the *y*‐axis varies considerably from (a–i). The number in the upper left corner of each figure stands for the difference between the bats’ first emergence time relative to sunset, and a negative number indicates that first emergence started before sunset

We plotted the light intensity of 17 initial emergence times at different light intensities and displayed the change trend of natural light intensity after the onset of emergence. We found that the higher the light intensity, the faster the light intensity decreased per unit time (Figure 3). With a few exceptions, normally, the higher the light intensity at first emergence, the earlier the first emergence of *V*. *sinensis*.

### 3.2 The impact factors of the mid‐emergence time of *V. sinensis*

We recorded 140 mid‐emergence events of *V*. *sinensis*, only two events occurred before sunset. The difference between mid‐emergence time and sunset ranged from −11 to 62 (23.478 ± 11.232) min. Here, the positive values meant mid‐emergence events happened after sunset. A significant correlation between the mid‐emergence (relative to sunset) and the first emergence (relative to sunset) (*r*: .943, *p* < .001) was observed. The light intensity of mid‐emergence events was approximately 0.6 to 400 (40.459 ± 68.719) lux. In addition, 91.43% (128/140) mid‐emergence events occurred when the light intensity was below 100 lux, and 42.86% (60/140) of mid‐emergence events occurred when the light intensity was below 10 lux.

**FIGURE 4:**
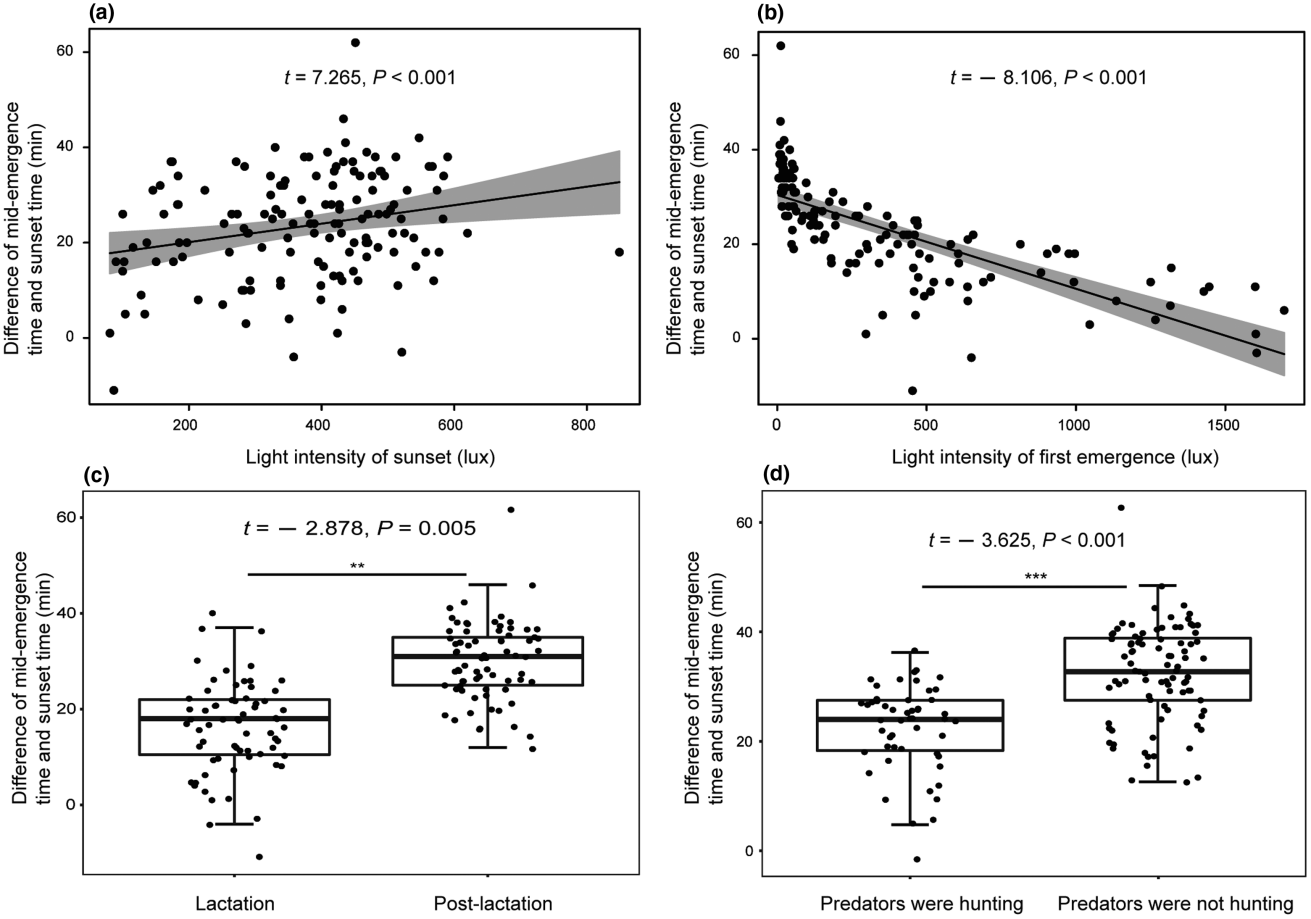
The relationships between differences (between sunset time and mid‐emergence time) and light density of sunset (a), and light density at first emergence (b), and reproductive status (c), and predators were hunting or not hunting (d). The gray area represents the 95% CI. **Means *p* < .01; ***Means *p* < .001

Our results indicated that the best‐fitting model of variation in mid‐emergence time used LIFE, LISS, LS, and PHNHM as the predictor variables (Table 3). Furthermore, model averaging revealed that the four predictor variables were also significantly associated with the mid‐emergence time (Table 4). First, differences between the mid‐emergence time and sunset time were significantly and negatively associated with light intensity of first emergence (*t* = −8.106, *p* < .001, Figure 4b). Second, there was a significant and positive correlation between the mid‐emergence time and light intensity at sunset (*t* = 7.265, *p* < .001; Figure 4a). Additionally, the LIFE and LS together had significant influence on mid‐emergence time (*t* = 3.425, *p* < .001, Table 4). Third, a significant difference in the mid‐emergence time was detected between lactation and postlactation (*t* = −2.878, *p* < .001; Figure 4c). During lactation, *V*. *sinensis* start emergence earlier than during postlactation (Figure 4c). Specifically, the mid‐emergence events started approximately −11 to 42 (16.269 ± 9.561) min after sunset during lactation. In contrast, the mid‐emergence events started about 0 to −62 (30.096 ± 8.158) min after sunset during the postlactation. Finally, the mid‐emergence time also had a significant difference depending on whether the predators were preying on bats (*t* = −3.625, *p* = .008) (Table 4). The results of the hierarchical partitioning analysis showed that the independent contributions of LIFE, LISS, LS, and PHNHM were 45.696%, 13.275%, 25.515%, and 15.513%, respectively.

**TABLE 3:** Candidate linear mixed models explaining the variation in mid‐emergence time of *Vespertilio sinensis* based on environmental factors (temperature, relative humidity, and light intensity), and biotic factors (reproductive stages and predation risk) at dusk

Model	Predictive variables	k	df	△AICc	*w_i_*
M1	LIFE, PHNHM, LS, LISS, LIFE*LS	5	6	0.000	0.363
M2	LIFE, PHNHM, LS, LISS	4	7	0.623	0.266
M3	LIFE, PHNHM, TME, LS, LISS, PHNHM*TME	2	5	2.819	0.089
M4	LIFE, PHNHM, TME, LS, LISS, LS*LISE, PHNHM*TME	5	8	2.857	0.087
M5	LIFE, PHNHM, TME, LS, LISS, LS*LISE	4	7	3.114	0.076
M6	LIFE, PHNHM, TME, LS, LISS	5	6	3.539	0.062
M7	LIFE, PHNHM, TME, LISS, PHNHM*TME	4	7	6.415	0.015
M8	LIFE, LS, LISS, LS*LISE	4	7	6.711	0.013
M9	LIFE, PHNHM, LS, LISS, LS* PHNHM	5	8	6.885	0.012
M10	LIFE, HME, PHNHM, LS, LISS, LS* PHNHM	3	6	8.951	0.004

Note

Models are ranked by Akaike’ s Information Criterion corrected for small sample sizes (AICc) values, from the most plausible model to the tenth most plausible model.

Abbreviations: LIFE, light intensity of first emergence of bats; LISS, light intensity of sunset; LIME, light intensity of mid‐emergence of bats; HME, humidity as mid‐emergence of bats; TME, temperature at mid‐emergence of bats; LS, lactation stages; PHNHM, predators were hunting or not hunting on bats at mid‐emergence of bats; ST, sunset time.

**TABLE 4:** The parameter estimates of the best‐supported (before and including the null model) linear mixed models describing the variation in mid‐emergence time of *Vespertilio sinensis*. The independent effects (IF) of factors on the mid‐emergence time of *Vespertilio sinensis* using hierarchical partitioning analysis are displayed in the last column

Variable	Estimate	SE	*t* value	*p*	95% CI	IF
(Intercept)	22.147	1.944	12.254	<.001	18.299, 25.991	—
LIFE	−0.015	0.002	−8.106	<.001	−0.019, −0.011	45.696
LS	−4.111	1.427	−2.878	.005	−6.934, −1.289	25.515
LISS	0.027	0.004	7.265	<.001	0.020, 0.035	13.275
PHNHM	−4.591	1.243	−3.625	<.001	−7.050, −2.133	15.513

### 3.3 The impact factors for the final return time of *V. sinensis*

We recorded 78 final return events of *V*. *sinensis*, and these events occurred approximately 7 to 79 (30.474 ± 15.560) min before sunrise. There were no significant differences in the final return time between 2019 and 2020 (*t* = 1.730, *p* = .093).

**FIGURE 5:**
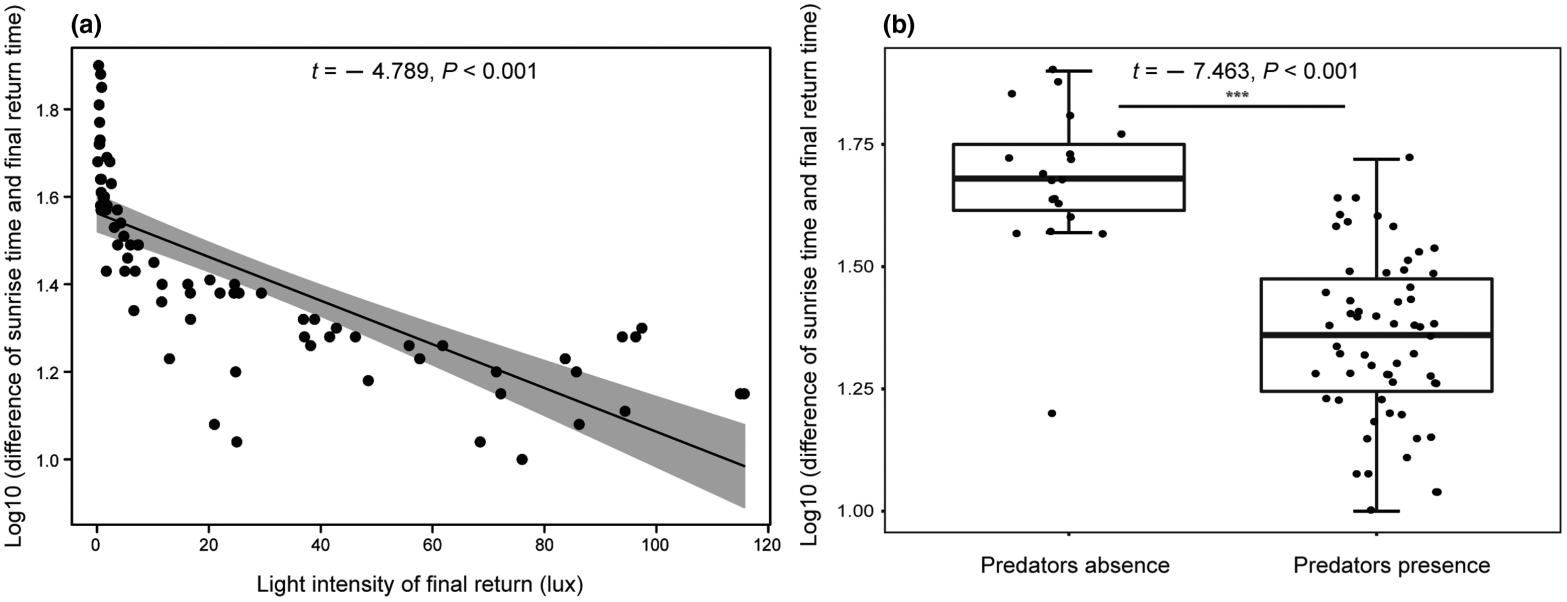
The relationships between differences (between final return time and sunrise time) and light density at final return (a), and predation status (b). The gray area represents the 95% CI. ***Means *p* < .001

Our results indicated that the best model of variation in the final return time used LIFR, LISR, TFR, HFR, LS, and PAPDA as the predictor variables (Table 5). The result of model fitting revealed that three predictive variables (LIFR, PAPDA, and interaction between LIFR and PAPDA) were significantly associated with the final return time (Table 6). The differences between sunrise and final return time were significantly and negatively associated with light intensity at final return (*t* = −4.789, *p* < .001, Figure 5a). The bats also returned to the roost later when predators were present (*t* = −7.463, *p* < .001; Figure 5b). We found that predators were often (60% of the records at dawn) present before the ending of the final return events at dawn. However, the number of successful predation events occurring at dawn was significantly less than at dusk (*Z* = −1.993, *p* = .046). The results of the hierarchical partitioning analysis showed that the independent contributions of LIFR and PAPDA were 60.427% and 39.573%.

**TABLE 5:** Candidate linear mixed models explaining the variation in final return time of *Vespertilio sinensis* based on environmental factors (temperature, relative humidity, and light intensity) and biotic factors (reproductive stages and predation risk) at dawn

Model	Predictive variables	k	df	△AICc	*w_i_*
M1	LIFR, PAPDA, LIFR*PAPDA	3	6	0.000	0.556
M2	LIFR, PAPDA	2	5	2.819	0.136
M3	LIFR, PAPDA, LS, LIFR*PAPDA, LIFR*LS	5	8	2.857	0.133
M4	LIFR, PAPDA, LS, LIFR*LS	4	7	3.114	0.117
M5	LIFR, TFR, PAPDA, LIFR*LS	4	7	6.415	0.022
M6	LIFR, PAPDA, LS, LIFR*PAPDA	4	7	6.711	0.019
M7	LIFR, TFR, PAPDA	3	6	8.951	0.006
M8	LIFR, PAPDA, LS	3	6	9.735	0.004
M9	LIFR, TFR, PAPDA, LS, LIFR*LS	5	8	11.920	0.001
M10	LIFR, TFR, PAPDA, LS, LIFR*PAPDA, LIFR*LS	6	9	12.093	0.001

Note

Models are ranked by Akaike’ s Information Criterion corrected for small sample sizes (AICc) values, from the most plausible model to the tenth most plausible model.

Abbreviations: LIFR, light intensity at final return of bats; LISR, light intensity of sunrise; HFR, humidity at final return of bats; TFR, temperature at final return of bats; LS, lactation stages; PAPDA, presence or absence of predators during dawn.

**TABLE 6:** The parameter estimates of the best‐supported (before and including the null model) linear mixed models describing the variation in final return time of *Vespertilio sinensis*. The independent effects (IF) of factors on the final return time of *Vespertilio sinensis* using hierarchical partitioning analysis are displayed in the last column

Variable	Estimate	SE	*t* value	*p*	95% CI	IF
(Intercept)	1.727	0.031	56.420	<.001	1.638, 1.798	—
LIFR	−0.022	0.004	−4.789	<.001	−0.034, −0.002	60.427
PAPAD	−0.248	0.033	−7.463	<.001	−0.313, −0.163	39.573
LIFR*PAPAD	0.018	0.004	3.933	<.001	0.009, 0.026	—

## 4 DISCUSSION

In our study, we found that light intensity at emergence, return, and sunset influenced the emergence and return times of *V*. *sinensis* in different ways, supporting our first hypothesis. Moreover, *V*. *sinensis* departed from the roost earlier regardless of the first emergence and mid‐emergence times during the lactation, supporting our second hypothesis. Finally, the mid‐emergence time of *V*. *sinensis* was earlier when predators were hunting, but the final return time was later when predators were present. This result was inconsistent with our third hypothesis.

Factors affecting an animal's energy status and the value of additional energy intake are major determinants of risk‐taking behavior (Brown, 1988; Caro, 2005; Lima, 1998). In general, the first emergence of bats occurs after sunset (Acharya et al., 2015; Arndt et al., 2018; Lee & McCracken, 2001; Welbergen, 2006) and return events occur before sunrise (Acharya et al., 2015; Lee & McCracken, 2001). During lactation, reproductive female bats would emerge before sunset because of the higher energy demand (Arndt et al., 2018; Lee & McCracken, 2001). This behavior has been noted in hoary bats, *Lasiurus cinereus* (Barclay, 1989), Mexican free‐tailed bats, *Tadarida brasiliensis* (Lee & McCracken, 2001), the gray‐headed flying fox, *Pteropus poliocephalus* (Welbergen, 2006), Daubenton's bats, *Myotis daubentonii* (Lučan, 2009), dawn nectar bats, *Eonycteris spelaea* (Acharya et al., 2015), and Indiana bats, *Myotis sodalis* (Arndt et al., 2018). Additionally, during lactation, reproductive female bats also return later to the day roost than during postlactation, such as in *T*. *brasiliensis* (Lee & McCracken, 2001) and Dawn nectar bats, *E*. *spelaea* (Acharya et al., 2015). Consistent with these previous studies, our results showed that lactation stages significantly influenced the first emergence and the mid‐emergence of *V*. *sinensis*. Specifically, 64.29% of emergence events started before sunset during lactation, while only 5.88% of emergence events started before sunset during postlactation. Bat activity had a positive relationship with the activity intensity of emerged insects (Salvarina et al., 2018), and peak activity of insects was always around dusk (Rydell et al., 1996). Thus, bats emerging from the day roost earlier gives bats the benefit of higher insect availability (Pavey et al., 2001). The higher energy demands may prompt bats to emerge from the roost earlier during the lactation. However, our results showed that lactation did not significantly affect the last return time of bats, which was not consistent with some previous studies stating that bats tended to return to the roost later during lactation (Lee & McCracken, 2001). This may be because *V*. *sinensis* emerged earlier to forage in order to meet the higher energy demand during the lactation. Additionally, the duration of bat emergence gradually decreased from lactation to nonlactation, which may be the result of some lactating females suckling or grooming their pups prior to emerging from the daytime roost (Lučan, 2009).

There are many benefits to an early start to night activity, but for bats, doing so in bright light may be a risky option. Large flocks of bats emerging from the day roost and returning to the day roost will attract the attention of diurnal avian predators (Fenton et al., 1994). Predation risk would affect many aspects of bats’ behavior, such as roost selection and foraging activity (Fenton et al., 1994; Lima & O’Keefe, 2013). Bats emerging around dusk before sunset under high light intensity would be exposed to higher predation risk than the bats emerging in true night under low light intensity. As for earlier emerging bats and later returning bats, both would be exposed to high predation risk from diurnal predators (Fenton et al., 1994). In this study, we found that the mid‐emergence time of *V*. *sinensis* was earlier when predators were hunting. This can be explained by the following reasons. First, bats tend to emerge significantly earlier from colonies with more individuals (Arndt et al., 2018; Fenton et al., 1994). There were more than 6000 individuals in this population. Therefore, here, the early emergence of bats in the presence of predators may be the result of the large population size. Second, the length of lactation is about 28 days in *V*. *sinensis* (Yin, 2020), and *V*. *sinensis* usually gives birth to twins in each litter (Jin et al., 2012), which may lead to more time spent nursing offspring at night. As a result, the short lactation duration and multiple offspring may cause high‐energy demands for *V*. *sinensis*. Thus, even though *V*. *sinensis* may suffer higher risk of predation, they may need to emerge from the roost earlier during the lactation due to the high‐energy demands. The hierarchical partitioning analysis also confirmed that lactation had a greater contribution to the mid‐emergence time and the first emergence time in *V*. *sinensis* than predation risk. Interestingly, predation risk only influenced the mid‐emergence time rather than the first emergence time in *V*. *sinensis*. This may be because *V*. *sinensis* had to emerge in quantity around the mid‐emergence time due to *F*. *amurensis* successfully preying on bats in dim light (light intensity below 10 lux); this also may reduce the risk of predation on individuals via the dilution effect of a large flock of bats (Wilkinson, 1985).

Bright light conditions may present a risky option for nocturnal animals, especially for bats. In this study, light intensity was the most important factor affecting the activity rhythm of *V*. *sinensis* (Figure 1). Specifically, differences between first emergence time and sunset time, and between mid‐emergence time and sunset time, were significantly and positively associated with light intensity at sunset (Figures 2a and 4a). These results showed that *V*. *sinensis* may adjust the emergence times based on sunset times. This may be helpful for avoiding *F*. *amurensis* for the following reasons. With the decrease in light intensity, the visual sensitivity of falcons decreases significantly (Fox et al., 1976). In our observations, when the light dimmed, it was harder for the Amur falcons to hunt, and if the bats did not begin to emerge, *F*. *amurensis* would give up waiting for bats. Therefore, it was clearly a safer behavioral strategy for bats to emerge from the roost under dim light. Additionally, bright light exerts inhibitory effects on the activity of bats (Gutierrez et al., 2014). However, here the opposite trends of the effect of light intensity of first emergence on the emergence time (Figures 2b and 4b) and of light intensity at final return on the final return time (Figure 5a) were observed. These findings may be due to the high‐energy demands in *V*. *sinensis* during lactation prompting the bats to depart from the roost earlier and return to the roost later despite the high light intensity. Additionally, bright light inhibited bat activity rhythms (Erkert, 2004), which may reflect the fact that emerging from the day roost early at dusk or returning to the roost late at dawn were not the best options for the bats.

Weather conditions may also affect the emergence behavior of bats (Erickson & West, 2002; Frick et al., 2012; Welbergen, 2008). For example, the relationship between temperature and the start of emergence of bats depended on summer climatic conditions; therefore, the influence of daily temperature on drought conditions may be different than in normal or unusually moist years (Frick et al., 2012). Below a critical minimum temperature, the foraging behavior of bats would be less beneficial than remaining in torpor (Avery, 1985). Drought conditions were associated with low insect abundance (Hawkins & Holyoak, 1998), and thus were associated with earlier emergence and displaying risker behavior (Frick et al., 2012). Our results indicated that temperature and humidity did not significantly influence emergence or return times of *V*. *sinensis*. This may because during our survey period in July to early September, the temperature was warm at this time, so the environmental temperatures did not have significant effects on *V*. *sinensis* during the monitoring period. Additionally, the rainfall was 672.7 and 792.8 mm in the study area in 2019 and 2020, respectively (Heilongjiang Provincial Government, 2021a; Liu & Wei, 2020), which was higher than the average annual precipitation (515.16 mm) in wet years (Zang et al., 2020). Moreover, according to an announcement by the local government, the summer precipitation in 2021 was also expected to be higher (Heilongjiang Provincial Government, 2021b). Since the increased precipitation could lead to the increase in arthropod biomass (Wilson et al., 2013), an increase in local arthropod biomass in summer in this area from 2019 to 2021 would be observed. Thus, the high abundance of local arthropod biomass may weaken the effects of temperature and humidity on activity rhythms of bats.

## 5 CONCLUSIONS

In summary, our study demonstrated that the emergence and return times of *V*. *sinensis* were affected by light intensity, reproductive status, and predation risk in a relatively complex pattern, indicating that the decisions concerning emergence and return of *V*. *sinensis* had a high degree of plasticity. Our results also highlighted that higher energy demands during lactation in bats may be more important than predation risk in the variation in activity rhythms. Future studies need to consider more factors, such as the distance between foraging sites and the bats’ roost, the number of foraging sites, and the intensity of insect activity and richness of insect species, to determine the impact of these factors on bat activity patterns.

## AUTHOR CONTRIBUTIONS

**Lei Feng:** Conceptualization (equal); Data curation (equal); Formal analysis (lead); Investigation (equal); Methodology (lead); Visualization (lead); Writing – original draft (lead). **Hexuan Qin:** Investigation (equal). **Jingjing Li:** Investigation (equal). **Xin Li:** Investigation (lead). **Jiang Feng:** Funding acquisition (equal); Resources (lead). **Tinglei Jiang:** Conceptualization (equal); Data curation (equal); Funding acquisition (lead); Project administration (lead); Supervision (lead); Writing – review & editing (lead).

## CONFLICT OF INTEREST

The authors declare that they have no conflicts of interest.

## Supporting information

Supplementary Material

Supplementary Material

Supplementary Material

Supplementary Material

## Data Availability

The data generated for this study are available at Dryad (https://doi.org/10.5061/dryad.0k6djhb2c).
