# Supplementary figures and images for: Extrinsic and intrinsic factors influencing the emergence and return of the Asian particolored bat Vespertilio sinensis to the summer roost
Source: Ecol Evol. 2022 May 13;12(5):e8890. doi: 10.1002/ece3.8890 (PMC9106590; doi:10.1002/ece3.8890)

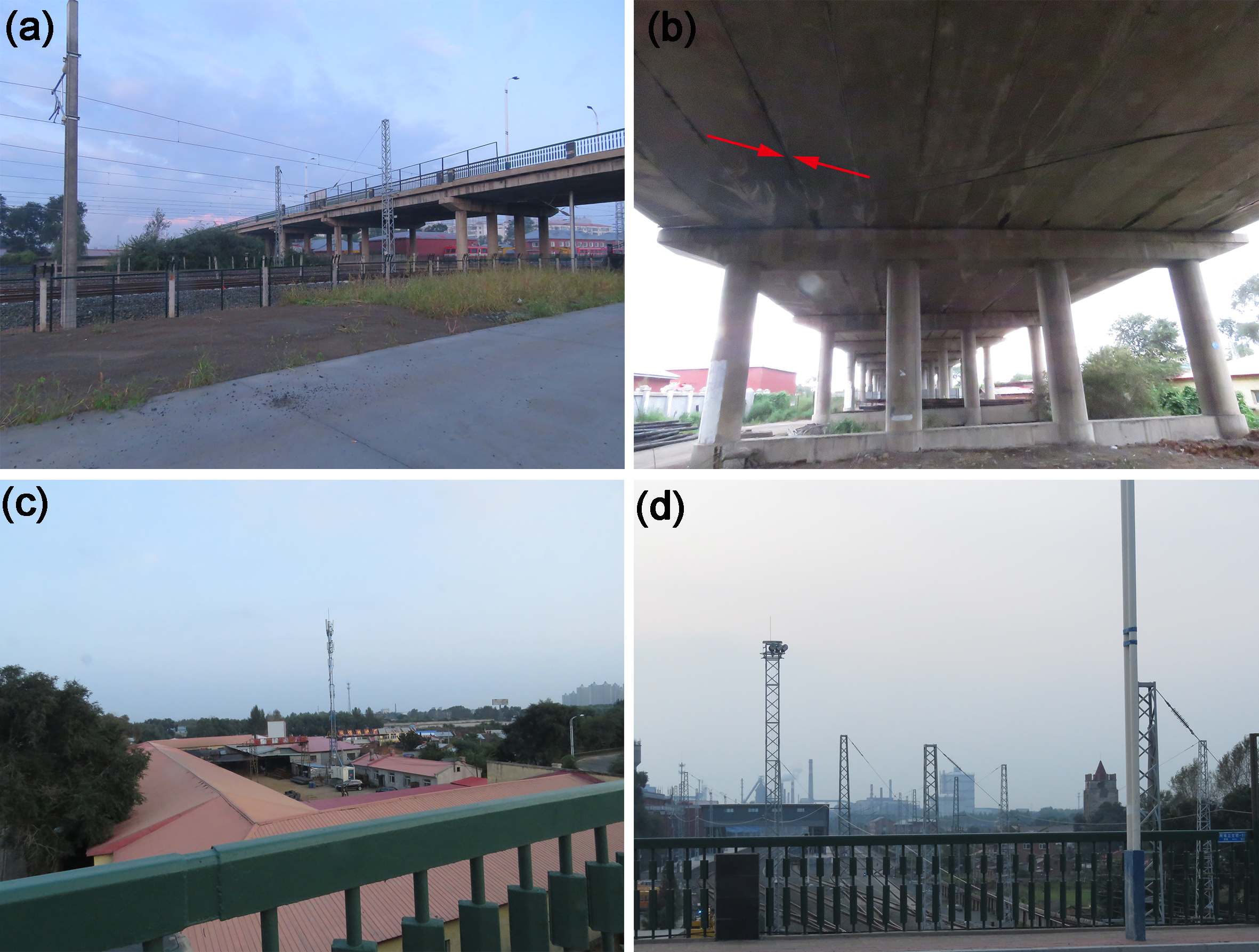

Supplement: Supplementary file 1 — Supplementary Material [file ECE3-12-e8890-s002.tif]

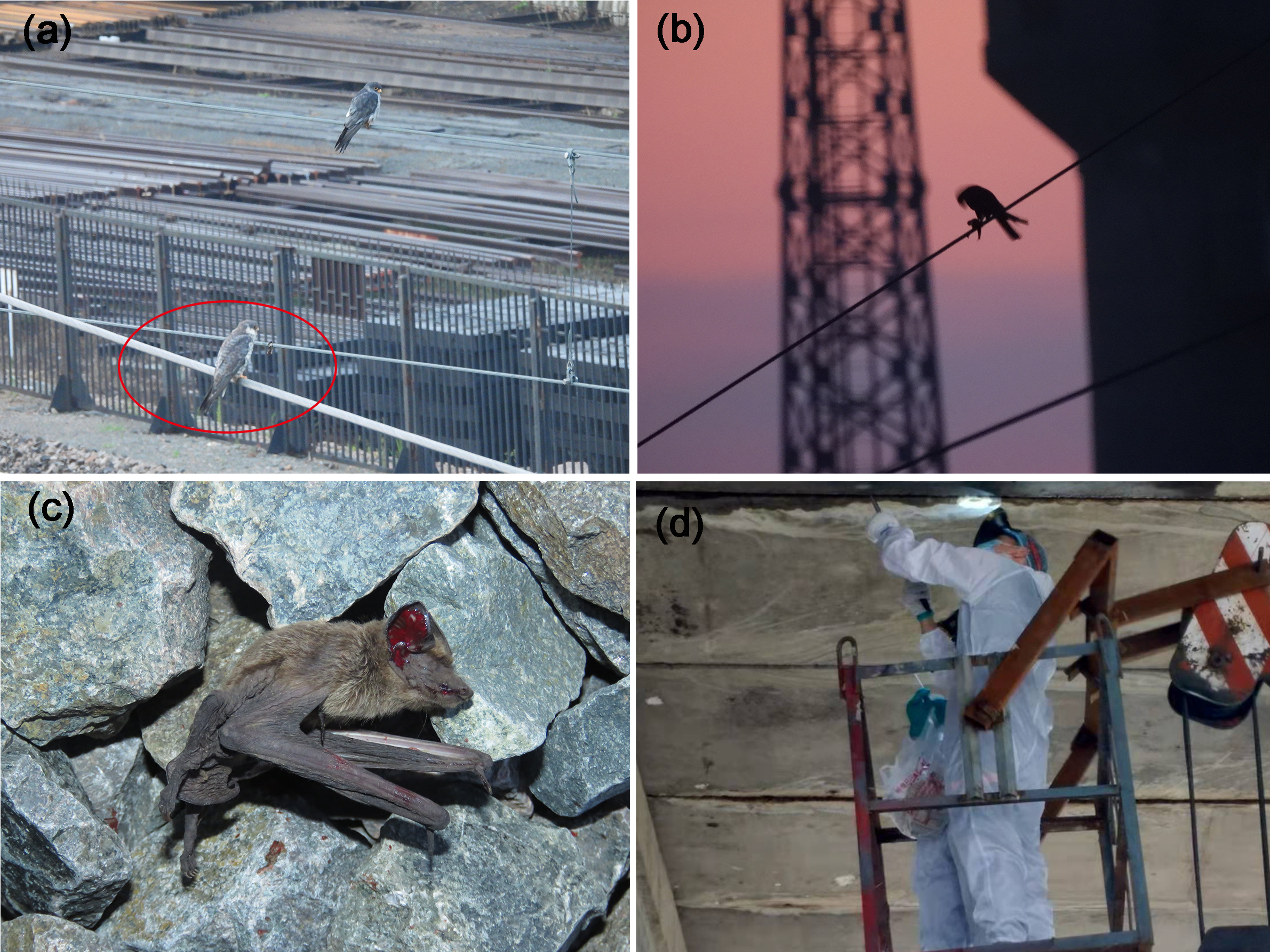

Supplement: Supplementary file 2 — Supplementary Material [file ECE3-12-e8890-s001.tif]

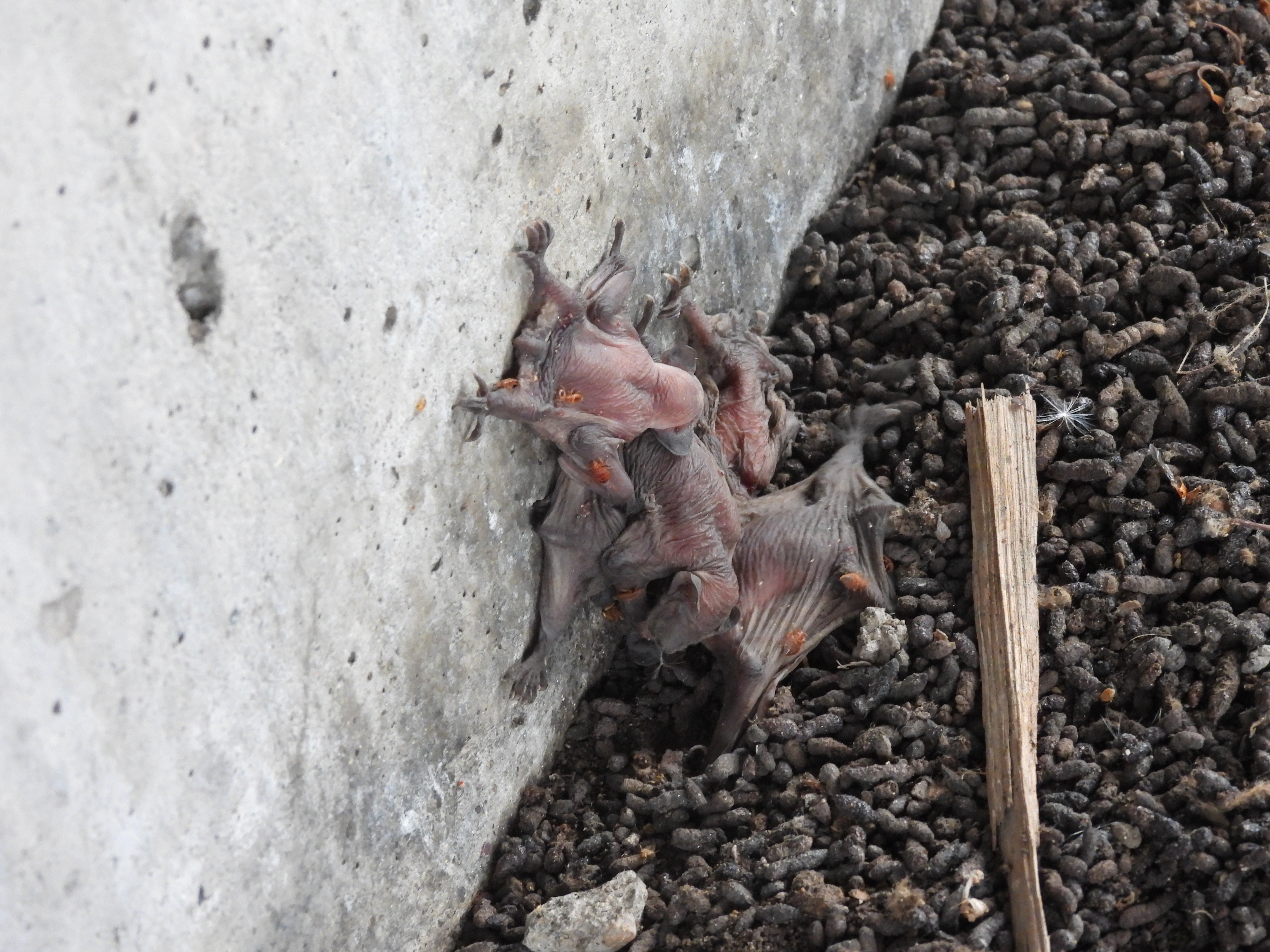

Supplement: Supplementary file 3 — Supplementary Material [file ECE3-12-e8890-s003.tif]
