# Supplementary material for: Extrinsic and intrinsic factors influencing the emergence and return of the Asian particolored bat Vespertilio sinensis to the summer roost
Source: Ecol Evol. 2022 May 13;12(5):e8890. doi: 10.1002/ece3.8890 (PMC9106590; doi:10.1002/ece3.8890)
Supplement: Supplementary file 4 — Supplementary Material [file ECE3-12-e8890-s004.docx]

APPENDIX 1


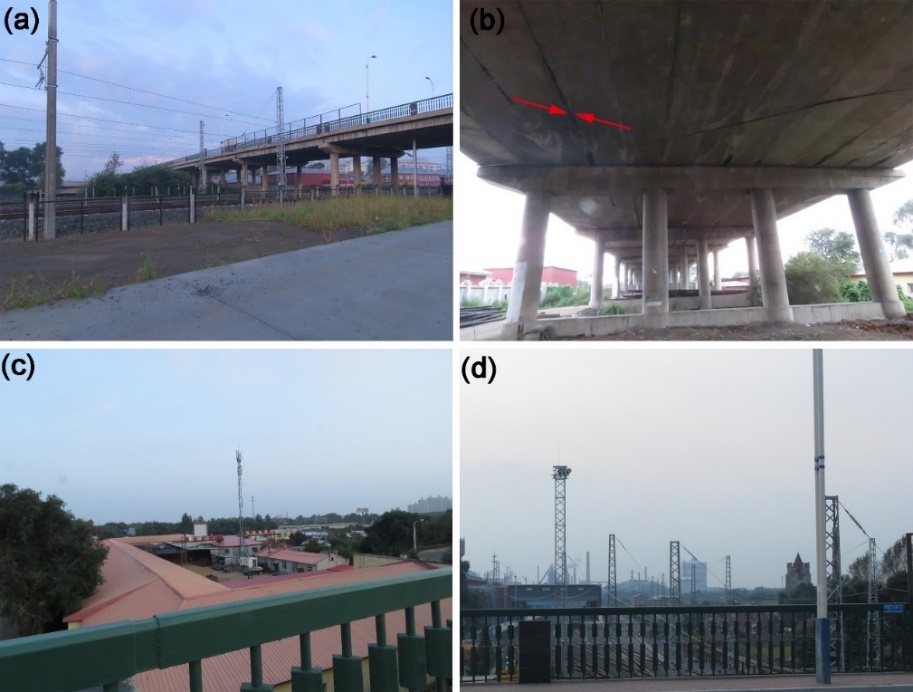


FIGURE A1 (a) Panoramic view of the overpass; (b) the crevices under the bridge where bats roost, where the red arrows point to a crevice; (c) the vegetation around the overpass; (d) the buildings around the overpass.


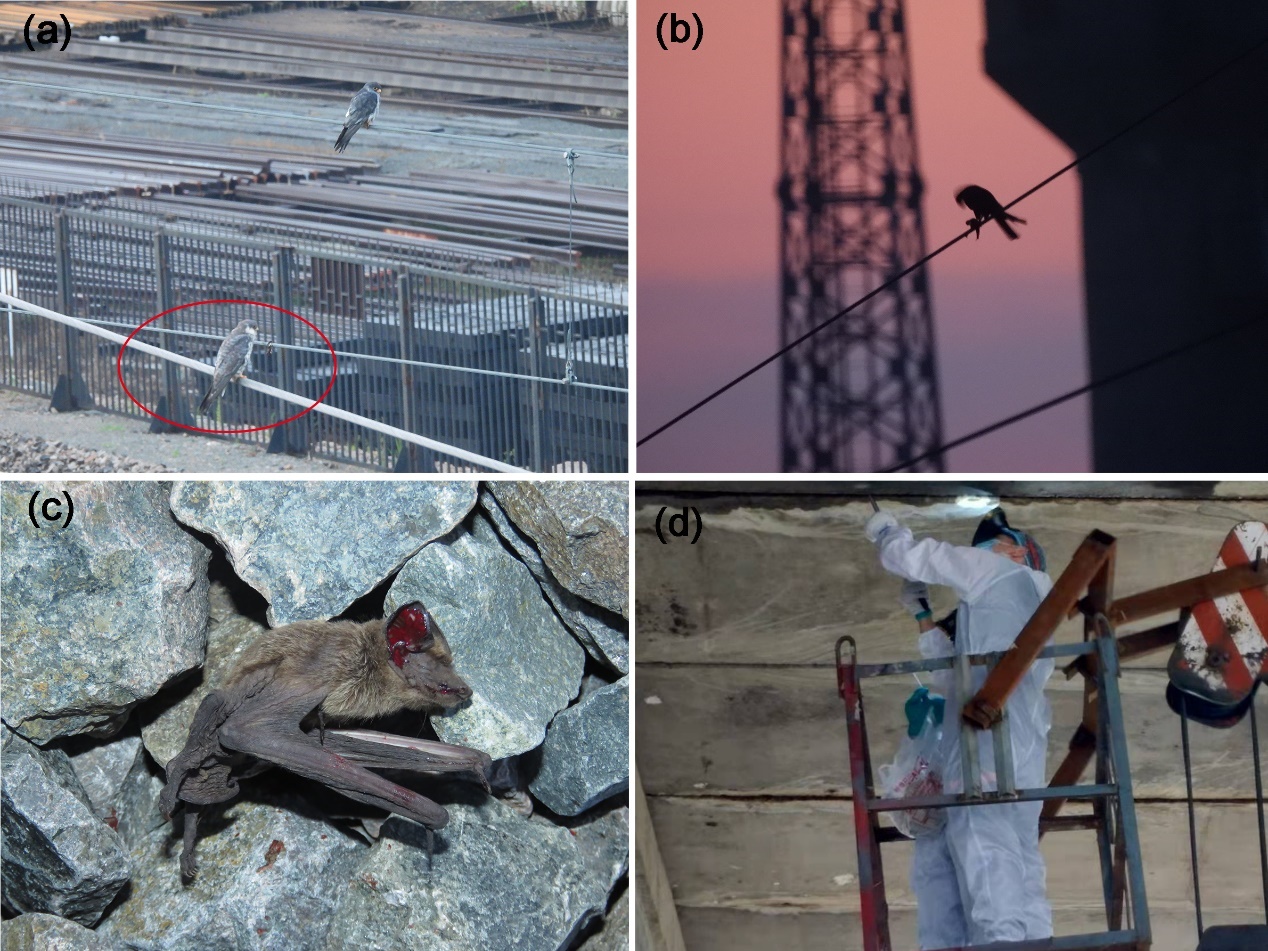


FIGURE A2 (a) Two Amur falcons waiting for emerged bats, where the red oval marks a female Amur falcon; (b) an Amur falcon and a hunted bat; (c) a hunted bat fallen on the ground; (d) the experimenter counting the bats that roosted in the crevices under the bridge.


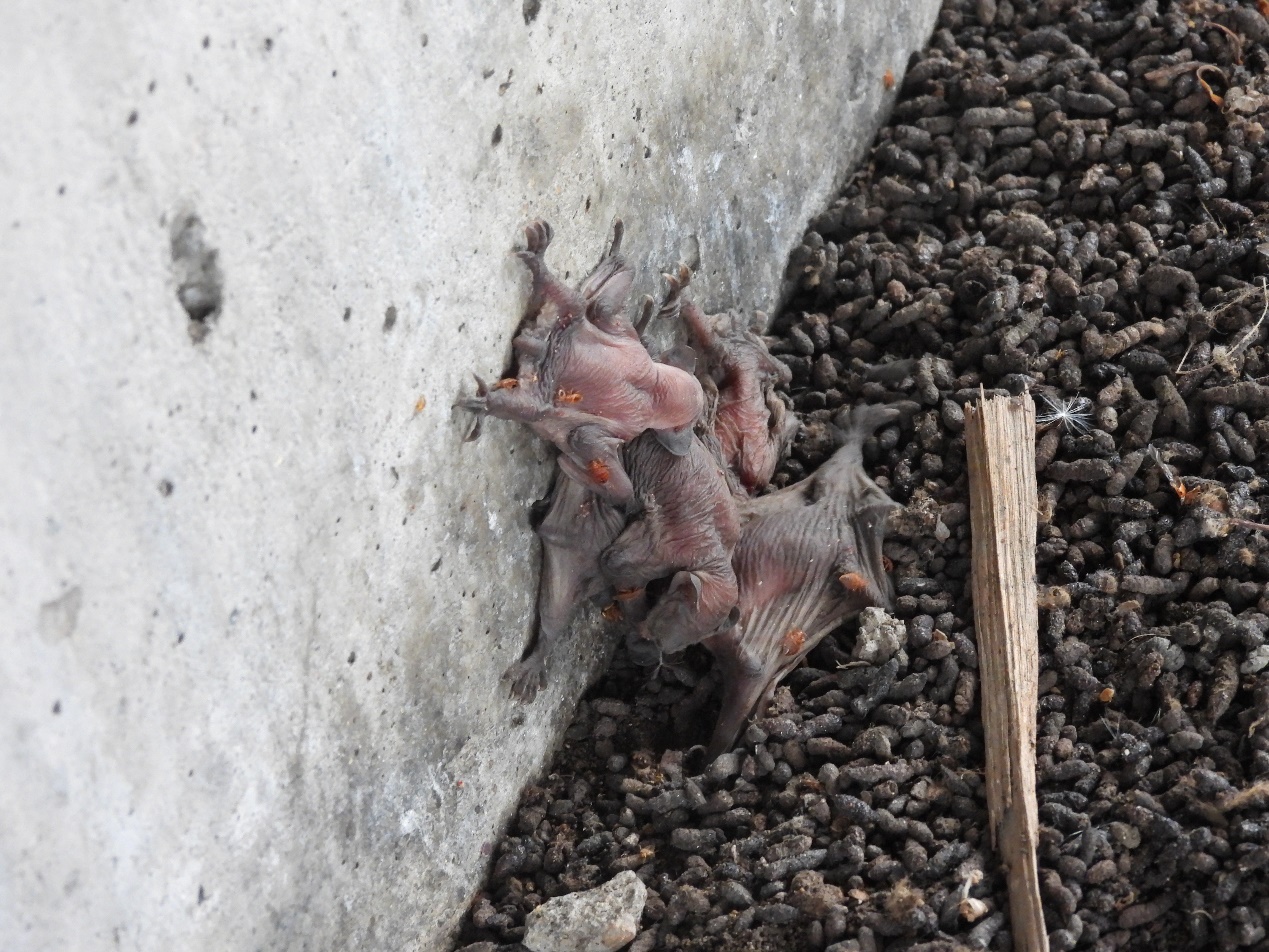


FIGURE A3 Juvenile bats that had fallen on the ground; parasites can be seen on the bodies of the juvenile bats.
